# Development of DNA Markers for Acute Hepatopancreatic Necrosis Disease Tolerance in *Litopenaeus vannamei* through a Genome-Wide Association Study

**DOI:** 10.3390/biology13090731

**Published:** 2024-09-18

**Authors:** Sukhuman Whankaew, Phassorn Suksri, Ammara Sinprasertporn, Jumroensri Thawonsuwan, Ponsit Sathapondecha

**Affiliations:** 1 Faculty of Technology and Community Development, Thaksin University, Phatthalung Campus, Phatthalung 93210, Thailand; sukhuman.wha@gmail.com; 2 Center for Genomics and Bioinformatics Research, Division of Biological Sciences, Faculty of Science, Prince of Songkla University, Hat Yai, Songkhla 90110, Thailand; phassorn.tua5122@gmail.com; 3 Songkhla Aquatic Animal Health Research and Development Center, Department of Fisheries, Songkhla 90110, Thailand; naphanang@gmail.com (A.S.); pjumroensri@gmail.com (J.T.)

**Keywords:** *Vibrio parahaemolyticus*, acute hepatopancreatic necrosis disease, DArT sequencing, genome-wide association study, shrimp

## Abstract

**Simple Summary:**

Acute hepatopancreatic necrosis disease (AHPND) is one of the most serious diseases, leading to massive levels of death in marine shrimp. To achieve sustainable prevention, molecular breeding is a promising approach that requires DNA markers for genetic selection. The aim of this study was to identify DNA markers associated with the AHPND-tolerant phenotype in *Litopenaeus vannamei* using DArT sequencing and a genome-wide association study. The post-larval shrimp were examined for infection with *Vibrio parahaemolyticus*, which causes AHPND (Vp_AHPND_), and examined for AHPND susceptibility and tolerance. DNA markers were identified in 93 individuals, including single-nucleotide polymorphisms (SNPs) and insertions/deletions (InDels). We found a number of filtered SNPs and InDels, while only four SNPs and 17 InDels were significantly associated with the trait of AHPND tolerance. This information could be useful as a genetic tool for the selection of AHPND-tolerant shrimp.

**Abstract:**

Shrimp aquaculture is facing a serious disease, acute hepatopancreatic necrosis disease (AHPND), caused by *Vibrio paraheamolyticus* (Vp_APHND_). For sustainable shrimp aquaculture, massive losses of shrimp infected with Vp_APHND_ must be prevented. Research and selection of shrimp tolerant to Vp_APHND_ infection is a sustainable approach to reducing the risk of AHPND. This study focused on the identification and development of potential DNA markers associated with AHPND using DArT sequencing (DArTSeq) and a genome-wide association study. Three populations of post-larval *Litopenaeus vannamei* were immersed in Vp_APHND_ to collect susceptible (D) and tolerant (S) samples. The 45 D and 48 S shrimp had their genotypes analyzed using DArTSeq. A total of 108,983 SNPs and 17,212 InDels were obtained from the DArTseq data, while the biallelic 516 SNPs and 2293 InDels were finally filtered with PIC < 0.1, MAF < 0.05, and a call rate ≥ 80%. The filtered variants were analyzed for their association with AHPND tolerance. Although there were no significantly associated SNPs and InDels above the Bonferroni correction threshold, candidate variants, four SNPs and 17 InDels corresponding to *p* < 0.01, were provided for further validation of the AHPND tolerance trait. The candidate SNPs are located on an exon of the zinc finger protein 239-like gene, an intron of an uncharacterized gene, and in intergenic regions. Most of the candidate InDels are in the intergenic regions, with fewer in the intronic and exonic regions. This study provides information on SNPs and InDels for white shrimp. These markers will support the variant database of shrimp and be useful in shrimp aquaculture for breeding selection.

## 1. Introduction

Pacific white shrimp (*Litopenaeus vannamei*) is one of the most economically important animals in several countries. However, in recent decades, acute hepatopancreatic necrosis disease (AHPND) or early mortality syndrome (EMS) has emerged in shrimp farming, leading to massive mortalities in shrimp farming and thus economic losses [1]. Vibrio parahaemolyticus was first identified as the causative agent of AHPND (Vp_AHPND_) [2]. There have been several attempts to control AHPND in shrimp farms, such as by using antibiotic treatment, pond management, and post-larval pathogen-free control, but they have not been successful so far [1]. To achieve sustainable control of AHPND, genetic selection is one of the most promising approaches to reducing the risks of pathogen infection in aquaculture by improving genetic accuracy [3].

Molecular breeding is one of the most accurate and precise approaches for selecting animals with the desired traits using molecular markers [4]. This involves the use of marker-assisted selection (MAS) with DNA markers obtained from quantitative trait loci (QTL) and/or genome-wide association study (GWAS) analyses [5]. Single-nucleotide polymorphisms (SNPs) and insertions/deletions (InDels) are the most common DNA markers used for MAS due to their high diversity and frequency in the genome of aquatic species to facilitate breeding selection and accelerate the discovery of genes related to economic traits, such as fast growth [6,7]. In shrimp, SNPs and candidate genes were identified using GWAS for *L. vannamei* body weight [8], white spot syndrome virus (WSSV) [9], and sex phenotype [10]. However, the identification of variants in shrimp tolerant to Vp_AHPND_ has not been thoroughly studied. For example, the estimation of shrimp tolerance to Vp_AHPND_ using twelve microsatellite markers was studied [11]. The use of 508 amplicons from targeted sequencing yielded 30 candidate SNPs related to AHPND resistance in white shrimp [12].

The development of high-throughput sequencing platforms has become a more powerful tool to dissect the genetic basis underlying the traits. In shrimp, association analysis of 2b-RAD sequencing data, together with gene-based genotyping from two populations, identified a novel growth-related DNA marker in *L. vannamei* [13]. Targeted amplicon sequencing was used to analyze genotypes to provide an SNP dataset of *L. vannamei* [14]. In addition, SNPs were developed from RNA sequencing data [15] and a 50K SNP chip [9] in *L. vannamei*. DArTSeq, a similar technique to ddRAD sequencing, has the advantage of reducing complexity by digesting methylated DNA sequences using restriction enzymes to the genome in a library preparation before undergoing Illumina sequencing [16]. It has been widely used as a genotyping and sequencing approach to obtain either silico DArT and DArT SNPs or SNPs and InDels [17]. A comparison of marker-associated traits using DArTseq and GBS indicated that some QTLs are commonly found in European winter wheat [18]. In addition, it is widely used to identify variants and subsequent analysis in QTL and/or GWAS [19,20,21]. It was also used to identify and develop SNPs in *Penaeus monodon* [22]. To date, there is no large-scale sequencing data used to investigate an association of AHPND tolerance in shrimp. Therefore, this study aimed to identify DNA markers using DArTSeq and analyzed the association of genotypes with AHPND tolerance in *L. vannamei*. This would provide a molecular basis for the prevention of disease and accelerate the breeding of shrimp that are tolerant to AHPND.

## 2. Materials and Methods

### 2.1. Animal Samples

Post-larval (PL15–20) *L. vannamei* were obtained from three different local shrimp farms in Songkhla province, Thailand. They were acclimatized to 30 ppt seawater at 25 °C for 3 days before use. Shrimp were fed twice daily with commercial feed pellets. The ethics for the use of animals were approved by the Institutional Animal Care and Use Committee, PSU (Ref. No. 97/2021), and the protocol was performed according to the regulation regarding the Animals for Scientific Purposes Act, B.E. 2558 (A.D. 2015), Thailand.

### 2.2. Bacterial Culture

Vp_AHPND_ J36 was streaked and cultured on TCBS agar at 37 °C overnight. A single colony was picked and cultured overnight in tryptic soy broth (TSB + 1.5% *w*/*v* NaCl) at 37 °C, 250 rpm. After overnight cultivation, the concentration was measured as cfu/mL by spreading the serially diluted culture on TSA containing 1.5% *w*/*v* NaCl.

### 2.3. Mortality Determination and Sample Collection of Shrimp Infected with Vp_AHPND_

Post-larval shrimp were reared in 1 L of 30 ppt seawater at a temperature of approximately 25 °C. The cultured seawater was changed every 4 days after the start of the experiment. Their lengths and weights were 0.94 ± 0.17 cm and 5.32 ± 2.14 mg, respectively. Approximately 150–200 individual shrimp from each population were used. They were immersed in 1 × 10^5^ cfu/mL Vp_AHPND_ J36. Dead or moribund shrimp (D) were collected every single day as AHPND-susceptible shrimp, while surviving shrimp (S) were harvested on day 14 as AHPND-tolerant shrimp. This was performed in triplicate. Whole bodies were extracted for their DNA using a conventional phenol/chloroform extraction method. The quality and quantity of DNA samples were evaluated by agarose gel electrophoresis and Nanodrop (Thermo Scientific, Waltham, MA, USA), respectively.

### 2.4. Sequencing and Variant Calling Analysis

The 93 DNA samples, including 48 D and 45 S shrimp, were analyzed for their genotypes by DArTSeq using the Illumina HiSeq 2500 platform with 138 bp single-end sequencing (Australia). The quality of the raw reads was evaluated using FASTQC [23], and adaptors, low-quality sequences, and sequence lengths less than 18 nt were removed using Cutadapt v. 4.4 [24]. To identify variants, the clean reads were mapped to the *L. vannamei* genome (RefSeq Accession No. GCF_003789085) using BWA version 0.7.17 [25] with default parameters. Mark duplication and data sorting were performed using MarkDuplicateSpark before variants were called using the GATK workflow [26]. The variants were hard-filtered with qual < 30, missing values < 20%, AC < 2, Info/DP < 10, QD < 2, and MQ < 40 using bcftools [27]. Filtered variants with polymorphic information content (PIC) < 0.1 and MAF < 0.05 were removed. The final variants were annotated using the ANNOVAR program (version 2020-06-07) [28]. The GFF file was used to construct the ANNOVAR annotation file and retrieve the gene location corresponding to the variant location and the up- and downstream positions of the variants.

For population structure analysis, the final filtered SNPs were used to analyze the dissimilarity index, and factorial analysis was performed using DARwin version 6.0 [29] and the clustering method via the Bayesian approach using STRUCTURE version 2.3.4 [30]. The SNP data were calculated for dissimilarity using the simple matching method with a bootstrap of 500 in the DARwin program. Subsequently, the dissimilarity result was used for factorial analysis and hierarchical clustering with the UPGMA method. To estimate the subpopulation (K), we set the burn-in period to 5000 and MCMC to 50,000. K was set from 1–8, with 20 iterations analyzed in the STRUCTURE program. The Ln P(D) values were then analyzed for K estimation using the delta K method [31] with the STRUCTURE HARVESTER program (version 0.6.93) [32] and the K optimum of 2 was determined. Finally, the population structure was analyzed with K = 2, a burn-in period of 50,000, and an MCMC of 500,000.

### 2.5. Genome-Wide Association Analysis for AHPND Tolerance

The filtered SNPs and InDels were used to analyze their association with the AHPND-tolerant phenotype in shrimp using GAPIT with a multi-locus mixed model (MLMM) [33]. The number of days that the shrimp were dead after Vp_AHPND_ infection was used as phenotypic data. The variants corresponding to a *p*-value < the Bonferroni correction threshold were selected. The explanation of genetic variation (PVE) was calculated according to [34]. The identification of the gene loci corresponding to the significant SNPs and InDels was performed with the ANNOVAR program. Subsequently, the identified gene loci from the shrimp genome were searched for gene names via the NCBI database. The ANNOVAR output also provided genes upstream and downstream of the SNP and InDel locations.

## 3. Results

### 3.1. Determination of Shrimp Mortality after Vp_AHPND_ Infection

**Figure 1:**
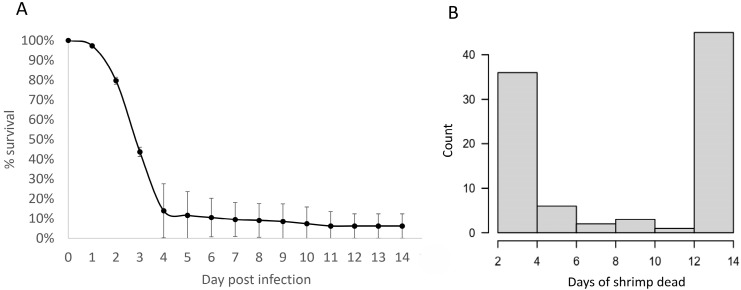
Shrimp mortality after Vp_AHPND_ infection. Post-larval shrimps were immersed in 1 *×* 10^5^ cfu/mL Vp_AHPND_, and the number of dead shrimps was recorded daily. The cumulative mortality of shrimp was presented in a line graph (**A**). Approximately 100–150 individuals were used in triplicate. Dots and error bars represent means and SEM, respectively. The number of dead shrimp was used to analyze their genotypes in each day, as shown (**B**).

Shrimp were infected with Vp_AHPND_ J36 and a number of dead samples were collected every day. The result indicated that most shrimp were dead or moribund 2–3 days after infection. Approximately 5% of shrimp survived until day 14 (Figure 1A). Among the dead group, most shrimp samples were dead on day 2 after infection, and only a few on later days (Figure 1B). Among the three populations, 48 dead (D) and 45 surviving (S) shrimp were selected and their sequences were analyzed using DArTSeq (Table S1).

### 3.2. DArTSeq and Genetic Diversity among the Three Populations of Tested Shrimp

**Figure 2:**
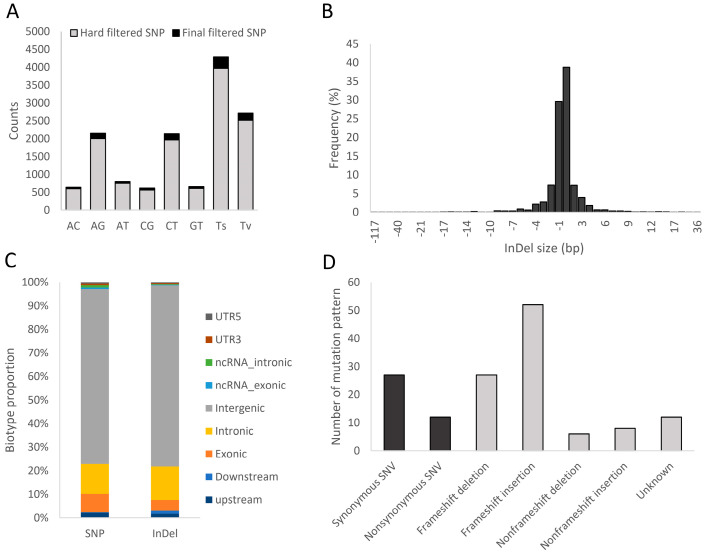
Summarized statistics of the filtered SNPs and InDels. The transition (Ts) and transversion (Tv) of the hard-filtered and final filtered SNPs were calculated and visualized in a bar graph (**A**). Distribution of the final filtered InDels’ gaps was counted (**B**). The biotypes of the final filtered SNPs and InDels were counted as percentages (**C**). For exonic variants, the number of mutational patterns was counted (**D**).

**Figure 3:**
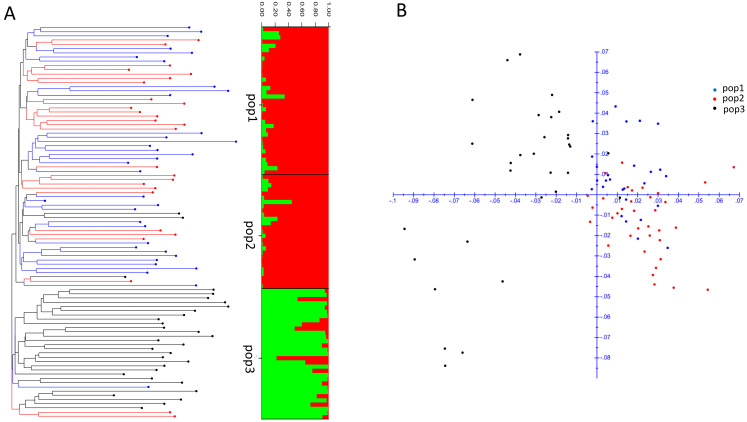
Clustering analysis of shrimp samples. Dendrogram of clustering analysis using the final filtered SNPs with UPGMA by DARwin and with Bayesian by STRUCTURE (**A**). The factorial analysis was performed based on the dissimilarity index by using DARwin (**B**). The blue, red, and black lines and dots represent data from population 1, 2, and 3, respectively. The red and green bars correspond to the average estimated cluster for all individuals in the population.

A total of 93 DNA samples from shrimp infected with Vp_AHPND_ were analyzed using DArTseq. From an average of 3,508,523 raw reads, an average of 3,508,465 clean reads with 285,948,354 bases were obtained (Supplementary Material File S1). These raw data were deposited as a sequence read archive at NCBI (BioProject No. PRJNA1137268). After variant calling, a total of 108,983 SNPs and 17,212 InDels were obtained, and hard filtration provided 53,083 SNPs and 15,557 InDels (Table 1). After a final filtering by discarding variants with PIC < 0.1, MAF < 0.05, and a call rate < 80%, only 516 SNPs and 2292 InDels remained. The details of the filtered SNPs and InDels and their functional annotation are shown in Supplementary Material Files S2 and S3, respectively. The transition/transversion ratios of hard-filtered SNPs and final filtered SNPs were 1.533 and 1.659, respectively (Table 1 and Figure 2A). The distribution of the final filtered InDel gaps showed that most InDels had a gap size of 1 nt (~67%) or 2–4 nt (~24.8%), with fewer having larger gaps (Figure 2B). The biotype of the SNPs and InDels was identified. The results revealed that the most SNPs and InDels were found in the intergenic regions (approximately 75%), with fewer in the intronic (12–14%) and exonic (4–7%) regions (Figure 2C). Moreover, the number of synonymous SNPs was greater than that of non-synonymous SNPs, while most filtered exonic InDels caused a frameshift mutation instead of a non-frameshift mutation (Figure 2D). Variant annotation is in the Supplementary Material Files S2 and S3. The final filtered SNPs were used to construct a dendrogram (Figure 3A) and to conduct factorial analysis (Figure 3B) based on a dissimilarity index. The subpopulation (K) was estimated using the delta K method and the optimum of K was 2 (Figure S2). The result indicated that the samples from two populations (pop1 and pop2) were more closely clustered and separate from another population (pop3) (Figure 3).

### 3.3. Association Analysis for AHPND Tolerance

**Figure 4:**
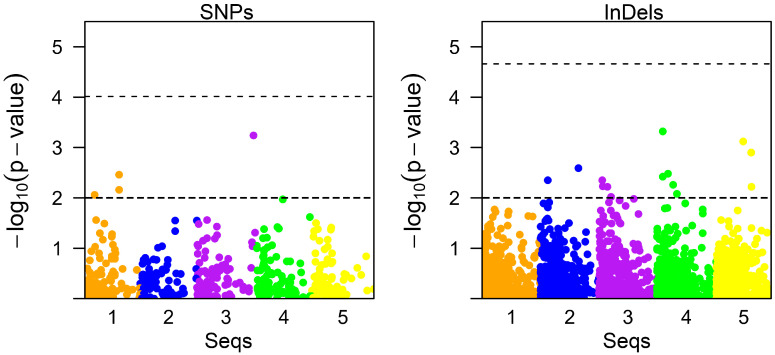
Manhattan plot of association analysis. The final filtered SNPs and InDels were used to perform association analysis with the AHPND-tolerant phenotype using the GAPIT program. The upper and lower dash lines represent the Bonferroni correction thresholds and a *p*-value of 0.01, respectively.

The final filtered SNPs and InDels were used to analyze the association with the AHPND-tolerant phenotype using the GAPIT program. Five models were evaluated in this study to select an appropriate model to reduce false positives. The Q-Q plots showed that the MLMM model had the least false positives compared to the other models for SNP data, while the MLMM, farmCPU, and BLINK models for InDels showed similar patterns (Figure S1). In addition, the heritability of the AHPND-tolerant trait was approximately 42% (Figure S3), indicating moderate heritability. In the association analysis, we found that there was no significant association of SNPs and InDels with the AHPND tolerance trait based on the Bonferroni correction threshold (9.6 × 10^−^^5^ and 2.14 × 10^−^^5^ for SNPs and InDels, respectively) (Figure 4). However, among the variants, four SNPs and 17 InDels had a *p*-value < 0.01 (Figure 4, Table 2 and Table 3), which could be considered for further validation in future studies. Since there were too many scaffolds of the *L. vannamei* genome, we grouped the genome scaffolds into a few sequences (Table 2 and Table 3).

Of the variants located in the intergenic regions of the *L. vannamei* genome, only SNP2501 was found on an exon of the zinc finger protein 239-like gene (ZNF239-like), while InDel3386 and InDel3811 were found on exonic regions of nascent polypeptide-associated complex subunit alpha, muscle-specific form-like (NACA), and probable phosphorylase b kinase regulatory subunit alpha (PHKA), respectively (Table 2 and Table 3). The phenotypic variation explained (PVE) of the candidate variants showed moderate values in the range of 5–13% (Table 2 and Table 3). In addition, extracellular protein-coding genes were mainly found near significant InDels such as vegetative cell wall protein gp1-like and proline-rich extensin-like protein EPR1, as well as some immune-responsive genes, including receptor-type guanylate cyclase Gyc76C-like and Ras GTPase-activating protein (Table 4).

## 4. Discussion

In shrimp aquaculture, AHPND is a serious disease that causes massive death within 3–7 days [1]. There have been several attempts to solve this important problem, such as by using antibiotics and/or probiotics, pond management, and post-larval stock control; however, successful control remains limited [1]. Molecular breeding is a promising tool to accelerate the development of desirable economic organisms [4]. In this study, AHPND-susceptible and -tolerant *L. vannamei* were sampled for genotyping analysis. Within the three populations, most shrimp were dead a few days after infection with Vp_AHPND_, and only a few shrimps survived (Figure 1). This was similar to the effect of Vp_AHPND_ on shrimp mortality, including *P. monodon* [35] and *L. vannamei* [10,36,37]. These shrimps were dead within 2–3 days post-infection, indicating the virulence of Vp_AHPND_ in shrimp. In addition, among the three populations of samples analyzed, we found that two populations are closely related and different from each other.

In recent studies, the genetic variation of shrimp has been investigated from several physiological aspects, including growth performance [38], ammonia tolerance [39], and pathogen infection [10]. These findings provided genetic markers from genotyping data generated by several types of DNA markers. For example, the genetic estimation of *V. parahaemolyticus* resistance was investigated using twelve microsatellite markers in *L. vannamei* [10]. The thousands of SNPs were used to analyze the genomic selection for growth and WSSV-resistant traits [38] and growth performance [8,13] in white shrimp. In this study, we obtained hundreds of SNPs and InDels from DArTSeq (Table 1). DArTSeq is a GBS technique that has been widely used in several organisms including crustaceans. Using double digestion with the restriction enzymes cleaving methylated DNA in a library preparation step before sequencing provided silico DArT and DArT SNP data, as well as analysis for SNPs and InDels from the sequencing data [16]. For example, thousands of SNPs were obtained after being analyzed from the DArTSeq data of *P. monodon* [22,40] and *Panulirus ornatus* [41]. However, a fairly limited number of SNPs and InDels might be obtained from strict filtering steps. Likewise, approximately 7000 SNPs were obtained and used in the GWAS analysis of the WSSV resistance trait in *L. vannamei* [9]. Some studies also had similar variant filtrations. In addition, The Ts/Tv ratio of the SNPs in this study was similar to other studies. For instance, the Ts/Tv of SNPs in *L. vannamei* was 1.91–1.97 under growth performance and WSSV infection using RNA sequencing [42], and 1.49–1.53 under high- and low-fecundity populations [38]. Most InDels had short gaps (1–3 bp), similar to InDels found in other organisms, including plants and animals [43]. In addition, the proportion of SNPs and InDels was dominant in the intergenic regions and lower in the intragenic regions (Figure 2C). Similarly, the number of SNPs and InDels found in intergenic regions was greater than in intragenic regions, including introns and coding sequences in other animals such as cattle [44].

GWAS is a powerful tool for association analysis between genotypes and phenotypes without breeding information [45]. In shrimp, candidate SNPs were identified that were associated with WSSV resistance and growth performance [9]. Similarly, 18 growth-related and 11 sex-related SNPs were identified in *Macrobrachium nipponense* [46]. In addition, the estimation of Vp_AHPND_ tolerance was performed with twelve microsatellite markers in white shrimp [11]. Considering the moderate heritability of the AHPND tolerance trait [11] and our heritability effect (Figure S3), the sample size in this study appeared small. In addition, there were no significant variants associated with the AHPND tolerance phenotype in relation to the Bonferroni correction threshold, but we found that the candidate four SNPs and 17 InDels were likely involved in the AHPND-tolerant phenotype in white shrimp. This would be useful for further validation with a larger sample size and application. Although there are a few GWAS analyses regarding the AHPND resistance trait in shrimp, our DArTseq data, as large-scale data, could be further used in meta-analysis studies. The meta-analysis of GWAS in combination with publicly independent GWAS results with similar traits could lead to an increase in the sample size, statistical power, and precision of the association analysis [47]. In addition, the association analysis in this study did not include adjustment of covariates such as the length and/or weight of the tested shrimp. This might result in a lower rate of actual association with the phenotype under consideration. However, our dataset could be useful for meta-analysis with a new dataset that adjusts for covariates such as shrimp weight using the recent method [48].

Most candidate variants exhibited moderate PVEs (Table 2 and Table 3). For example, some SNPs and InDels were located in the immune-responsive genes, including ZNF239-like, PHKA, and NACA, corresponding to a %PVE of 7.07%, 10.73%, and 14.42%, respectively. This suggests that polygenic involvement, including the candidate variants, has an impact on the AHPND tolerance trait. ZNF plays a role as a transcription factor that regulates several physiological processes, including cancer development [49] and the immune response [50]. PHKA is involved in the control of carbohydrate metabolism, particularly in glycan storage [51]. In shrimp, the increase in carbohydrate metabolism is triggered by pathogen infection [52]. NACA is a transcriptional cofactor that regulates AP-1 transcription, which is involved in cell differentiation [53]. It has been shown that the *EsNACA* expression was upregulated after infection with *Vibrio anguillarum* in a crab, *Eriocheir sinensis* [54]. These may indicate that the variants found on these genes have the potential to influence gene function, leading to a physiological change in AHPND tolerance in shrimp. The candidate SNPs and InDels identified in this study need to be further validated for AHPND tolerance in new shrimp populations. Expression analysis of the SNPs and InDels and their respective genes, as well as the neighboring genes located near the SNPs and InDels, would reveal crucial functions for the AHPND tolerance phenotype in shrimp. The validated ones will be used for the selection of AHPND-tolerant breeding broodstock to produce AHPND-tolerant offspring. This study provides information on genetic variants and potentially associated SNPs and InDels for the AHPND-tolerant trait in white shrimp, which could be useful for shrimp breeding programs in the future.

## 5. Conclusions

In this study, we conducted and collected *L. vannamei* samples from three populations that were susceptible and tolerant to Vp_AHPND_ infection. Using DArT sequencing, the polymorphic variants, SNPs and InDels, were obtained, and only four candidate SNPs and 17 InDels were selected from the AHPND-tolerant shrimp. This study provided information about variants with AHPND tolerance in white shrimp.

## Figures and Tables

**Table 1:** Summary of the variants analyzed from the experimental shrimp.

Description	SNPs	InDels
Total variants	108,983	17,212
Hard-filtered variants	53,083	15,557
Final filtered variants	516	2292

**Table 2:** The statistics of the associated SNPs to the AHPND-tolerant phenotype.

SNP Id	Biotype	Genome Accession No.	Position	*p*-Value	MAF	Effect	PVE (%)	Gene Name
SNP2515 C > A	Intergenic	NW_020869786.1	872,951	0.0022	0.12	−3.09	6.42%	-
SNP3827 A > C	Intergenic	NW_020870527.1	844,041	0.0033	0.19	−2.31	6.43%	-
SNP6128 A > T	Intronic	NW_020872696.1	875,105	0.0040	0.05	−3.72	9.29%	Uncharacterized (XM_027350909)
SNP2501 T > C	Exonic (Synonymous)	NW_020869780.1	528,035	0.0097	0.18	−2.14	7.07%	Zinc finger protein 239-like (XM_027364481)

**Table 3:** The statistics of the associated InDels to the AHPND-tolerant phenotype.

Candidate Markers	Ins/Del Gap (nt)	Biotype	Accession No.	Position	*p*-Value	MAF	Effect	PVE (%)	Gene Name
Indel0773	Ins 2	Intergenic	NW_020868718.1	1,222,723	2.4 × 10^−4^	0.17	−2.53	9.88%	-
Indel3486	Del 1	Intergenic	NW_020870028.1	97,508	4.8 × 10^−4^	0.31	−2.17	4.78%	-
Indel4880	Ins 2	Intergenic	NW_020870775.1	455,677	7.5 × 10^−4^	0.11	−2.87	13.25%	-
Indel4460	Del 1	Intergenic	NW_020870542.1	589,428	1 × 10^−3^	0.17	−2.32	4.83%	-
Indel4461	Ins 1	Intergenic	NW_020870542.1	589,433	1 × 10^−3^	0.17	−2.32	5.19%	-
Indel1142	Ins 1	Intergenic	NW_020868919.1	635,311	2.5 × 10^−3^	0.33	−1.72	5.26%	-
Indel3675	Ins 1	Intergenic	NW_020870115.1	185,999	3.3 × 10^−3^	0.065	3.11	8.86%	-
Indel3485	Ins 1	Intergenic	NW_020870028.1	97,483	3.8 × 10^−3^	0.31	−1.79	2.97%	-
Indel2533	Ins 1	Intergenic	NW_020869537.1	56,216	3.8 × 10^−3^	0.20	2.09	3.83%	-
Indel3811	Ins 1	Intergenic	NW_020870177.1	120,506	4.5 × 10^−3^	0.12	−2.45	10.66%	-
Indel0506	Del 1	Intronic	NW_020868577.1	271,787	5.5 × 10^−3^	0.065	2.93	10.73%	probable phosphorylase b kinase regulatory subunit alpha (XM_027376870)
Indel2406	Ins 1	Intergenic	NW_020869477.1	65,359	5.9 × 10^−3^	0.27	−1.73	5.06%	-
Indel3386	Del 1	Exonic (Frameshift deletion)	NW_020869981.1	594,057	5.9 × 10^−3^	0.11	−2.42	14.42%	nascent polypeptide-associated complex subunit alpha, muscle-specific form-like(XM_027366427)
Indel6073	Del 1	Intergenic	NW_020871916.1	143,341	6 × 10^−3^	0.65	2.95	11.85%	-
Indel6600	Ins 6	Intergenic	NW_020872438.1	335,379	8.2 × 10^−3^	0.19	−1.88	5.92%	-
Indel2592	Ins 1	Intergenic	NW_020869574.1	204,883	9.5 × 10^−3^	0.30	−1.68	8.24%	-
Indel2590	Del 3	Intergenic	NW_020869574.1	204,798	9.6 × 10^−3^	0.26	−1.87	2.95%	-

**Table 4:** Neighboring gene distance to the significant variants.

Candidate Markers	Distance (kb)	Gene Name (RefSeq Accession No.)	Function
SNP2515	Up 169.9	uncharacterized LOC113812673 (XM_027364589)	-
	Down 95.7	uncharacterized LOC113812685 (XM_027364609)	-
SNP3827	Up 357.7	vegetative cell wall protein gp1-like (XM_027371696)	Extracellular region
Indel0773	Up 64.4	proline-rich extensin-like protein EPR1 (XM_027353909)	Extracellular region
	Down 70.7	uncharacterized LOC113803169 (XM_027353894)	-
Indel3486	Down114.7	Zinc finger protein 749-like (XM_027366949)	Transcription
Indel4880	Up 29.2	Mucin-2-like (XM_027374070)	Extracellular region
Indel4460	Up 128	Receptor-type guanylate cyclase Gyc76C-like (XM_027371820)	Immune response
Indel4461	Up 128	Receptor-type guanylate cyclase Gyc76C-like (XM_027371820)	Immune response
Indel1142	Up 15.5	probable Ras GTPase-activating protein (XM_027355753)	Tumorigenesis
	Down 90.5	uncharacterized LOC113804845 (XM_027355756)	-
Indel3675	Up 50.2	putative neural-cadherin 2 (XM_027367808)	Cell-cell adhesion
Indel3485	Down 114.8	zinc finger protein 749-like (XM_027366949)	Transcription
Indel2533	Up 30.5	prolyl 4-hydroxylase subunit alpha-1-like (XM_027362215)	Collagen synthesis
	Down 190.4	basic salivary proline-rich protein 2-like (XM_027362206)	Extracellular region
Indel3811	Up 70.9	nephrin-like (XM_027368425)	Secretory system
Indel2406	Down 100.5	uncharacterized histidine-rich protein DDB_G0274557-like (XM_027361657)	-
Indel6073	Up 77.9	Trnal-cag_17	Translation
	Down 10.2	cell wall protein DAN4-like (XM_027379940)	Extracellular region
Indel6600	Up 190.5	cell surface glycoprotein 1-like (XM_027381906)	Extracellular region
Indel2592	Up 80.9	Trnaf-gaa_48	Translation
	Down 49.9	uncharacterized LOC113810924 (XM_027362580)	-
Indel2590	Up 80.9	Trnaf-gaa_48	Translation
	Down 49.9	uncharacterized LOC113810924 (XM_027362580)	-

## Data Availability

The DArT sequencing data of 93 shrimp were deposited at the NCBI and available in BioProject No. PRJNA1137268.

## Supplementary Materials

The supporting information can be downloaded at: https://www.mdpi.com/article/10.3390/biology13090731/s1. File S1: Information of the DArT sequencing data; File S2: The final filtered SNPs information; File S3: The final filtered InDels information; Table S1: Number of dead shrimp selected for use in this study; Figure S1: Q-Q plots of the five models’ SNPs and InDels. Figure S2: K estimation using Delta K analysis; Figure S3: Heritability of AHPND tolerance analyzed using the GAPIT program.
