# Supplementary material for: Development of DNA Markers for Acute Hepatopancreatic Necrosis Disease Tolerance in Litopenaeus vannamei through a Genome-Wide Association Study
Source: Biology (Basel). 2024 Sep 18;13(9):731. doi: 10.3390/biology13090731 (PMC11429464; doi:10.3390/biology13090731)
Supplement: Supplementary file 1 [file biology-13-00731-s001.zip › biology-3169780-supplementary/Supplementary Materials.pdf]

Supplementary Materials

Table S1 Number of dead shrimp selected to use in this study.

Day after infection	Number of shrimp dead			Total
	Population 1	Population 2	Population 3	
2	9	4	4	17
3	9	4	2	15
4	0	2	2	4
5	2	3	0	5
6	1	0	0	1
7	0	2	0	2
9	1	0	0	1
10	2	0	0	2
11	1	0	0	1
Survived	10	12	23	45

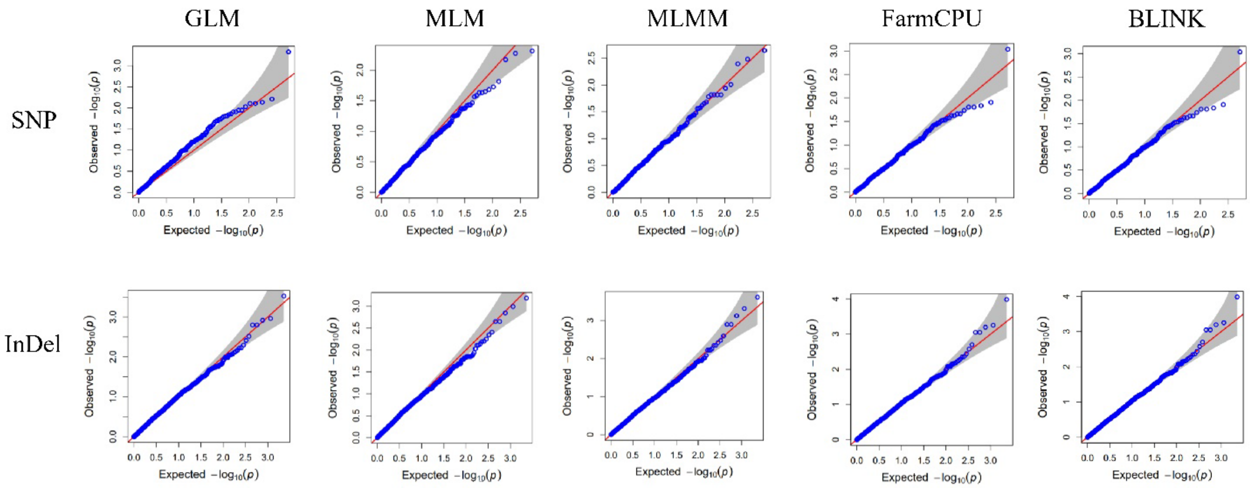

Figure S1. Q-Q plots of the five models' SNPs and InDels.

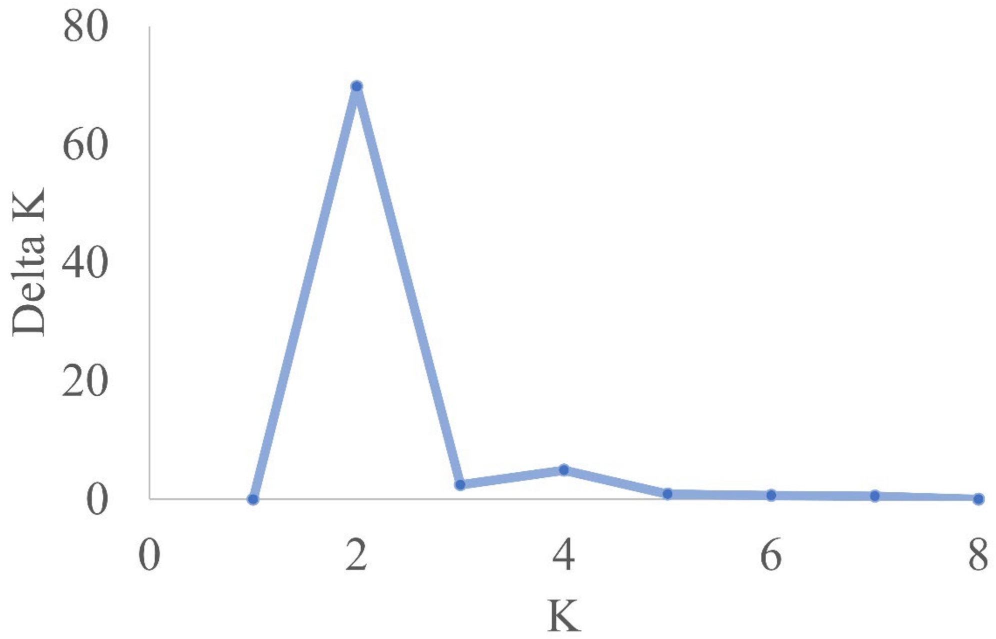

Figure S2: K estimation using Delta K analysis.

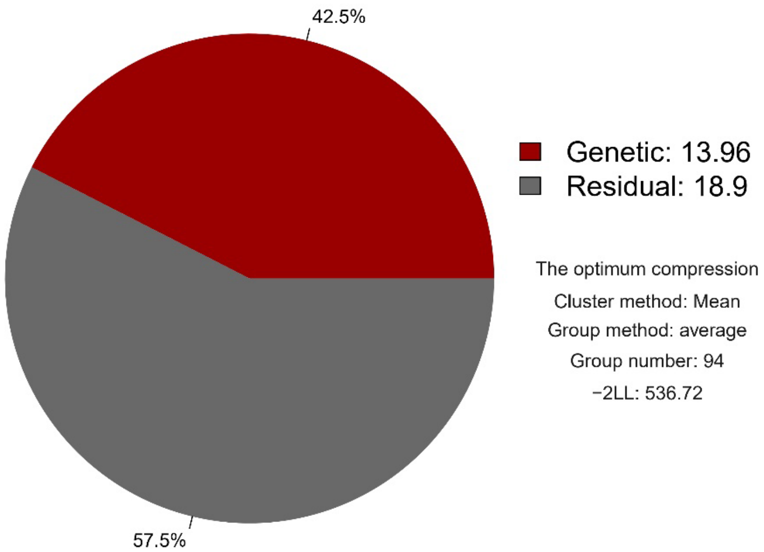

Figure S3: Heritability of AHPND tolerance analyzed using the GAPIT program.
